# Understanding the Potential Drivers for Respiratory Syncytial Virus Rebound During the Coronavirus Disease 2019 Pandemic

**DOI:** 10.1093/infdis/jiab606

**Published:** 2022-01-14

**Authors:** You Li, Xin Wang, Bingbing Cong, Shuyu Deng, Daniel R Feikin, Harish Nair

**Affiliations:** 1 School of Public Health, Nanjing Medical University, Nanjing, China; 2 Centre for Global Health, Usher Institute, University of Edinburgh, Edinburgh, United Kingdom; 3 Department of Immunizations, Vaccines, and Biologicals, World Health Organization, Geneva, Switzerland

**Keywords:** respiratory syncytial virus, pandemic, seasonality, COVID-19, nonpharmaceutical intervention, temperature, humidity, wind speed, school, susceptibility

## Abstract

Nonpharmaceutical interventions (NPIs) were widely introduced to combat the coronavirus disease 2019 (COVID-19) pandemic. These interventions also likely led to substantially reduced activity of respiratory syncytial virus (RSV). From late 2020, some countries observed out-of-season RSV epidemics. Here, we analyzed the role of NPIs, population mobility, climate, and severe acute respiratory syndrome coronavirus 2 circulation in RSV rebound through a time-to-event analysis across 18 countries. Full (re)opening of schools was associated with an increased risk for RSV rebound (hazard ratio [HR], 23.29 [95% confidence interval {CI}, 1.09–495.84]); every 5°C increase in temperature was associated with a decreased risk (HR, 0.63 [95% CI, .40–.99]). There was an increasing trend in the risk for RSV rebound over time, highlighting the role of increased population susceptibility. No other factors were found to be statistically significant. Further analysis suggests that increasing population susceptibility and full (re)opening of schools could both override the countereffect of high temperatures, which explains the out-of-season RSV epidemics during the COVID-19 pandemic.

## Teaser

Increasing population susceptibility and full (re)opening of schools have driven respiratory syncytial virus rebound during the COVID-19 pandemic, overriding the effect of temperature.

Respiratory syncytial virus (RSV) is the most common pathogen that causes hospitalization for pneumonia and bronchiolitis among young children globally [1–3]. RSV seasonal epidemics occur annually in most parts of the world and typically in autumn/winter in temperate regions [4]. Understanding RSV seasonality has important implications for healthcare services planning and immunization strategies, as well as recruitment for clinical trials of RSV prevention and treatment.

Following the onset of the coronavirus disease 2019 (COVID-19) pandemic in early 2020, nonpharmaceutical interventions (NPIs) were widely enforced by countries to reduce the spread of the severe acute respiratory syndrome coronavirus 2 (SARS-CoV-2). These interventions also likely resulted in substantial reduction in the circulation of RSV during its typical autumn/winter season in both the northern [5–11] and southern [12–15] hemispheres in 2020. Interestingly, some countries observed delayed out-of-season RSV rebound since late 2020, while other countries have not yet observed any RSV epidemics [5–7, 10, 11, 13, 15–17]. The underlying drivers for RSV rebound in some settings remain unknown. While the relaxation of NPIs can be an important driver [18], other factors such as climate [4, 19] and the possible viral interactions [20–22] could have also played a role in both RSV suppression and subsequent rebound. In this study, we sought to disentangle the role of these factors in RSV rebound through a time-to-event analysis among 18 countries.

## METHODS

### Study Design

#### Overview

This was a multicountry longitudinal observational study. The outcome of interest was the occurrence of RSV rebound since the onset of the COVID-19 pandemic. The exposures of interest included school opening status, population mobility, ban on international arrivals, COVID-19 notification rate, and meteorological factors. Eighteen countries (Australia, Belgium, Canada, Chile, Denmark, England, France, Iceland, Ireland, Japan, Netherlands, New Zealand, Paraguay, Portugal, Slovenia, South Korea, Spain, Sweden) with available data on both RSV activity (between 2019 and 2021) and exposures of interest were selected (Supplementary Table 1). We followed the Strengthening the Reporting of Observational Studies in Epidemiology (STROBE) guidelines for the reporting of our study (Supplementary Appendix).

#### Outcome

**Figure 1.**
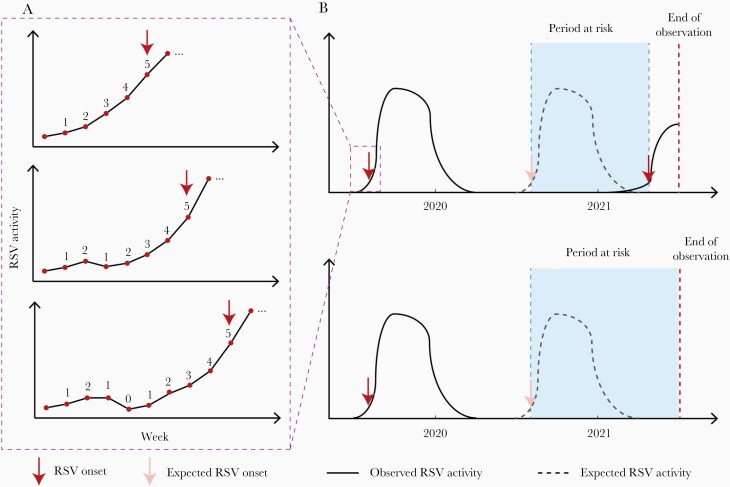
Schematic figure of the study design. *A*, Definition of respiratory syncytial virus (RSV) onset. The numbers next to the dots denote the difference in weeks between the number of weeks with increased RSV activity and the number of weeks with nonincreased RSV activity (ie, “net increasing weeks”). *B*, Definition of period at risk for RSV rebound.

Data on RSV weekly activity between 2019 and 2021 were accessed from national/regional viral surveillance reports identified through several previous works on RSV seasonality [4, 5, 20]; detailed data sources for RSV are available in Supplementary Table 1). For each RSV season, the season onset was defined based on whether an increasing trend in weekly reported RSV cases was observed. An increasing trend was confirmed when the number of increasing weeks exceeded the number of nonincreasing weeks by 5 (ie, 5 net increasing weeks) in any given intervals (Figure 1A). For example, if 5 consecutive increasing weeks was observed, then the RSV onset would be defined as the fifth increasing week; if 1 nonincreasing week was observed among several increasing weeks, then the RSV onset would not be confirmed until the sixth increasing week (so that the number of net increasing week is 6 – 1 = 5). The method for defining RSV season onset used in this study had several advantages compared with other existing methods. First, this method was based on short-term trend in positive tests and therefore was relatively less affected by varying testing practice over time (eg, before and during the COVID-19 pandemic) and among countries. Second, this method was not dependent on annual RSV data and could be used prospectively for detecting RSV season onset for timely response. Third, this method did not require the number of negative tests that were not available in some countries.

RSV rebound was defined as the first RSV onset that occurred after the expected RSV season onset during the COVID-19 pandemic. For each country, the period at risk for RSV rebound started from the expected onset of the first RSV season since the beginning of the COVID-19 pandemic, denoted as week 0, based on the timing of its last prepandemic RSV season onset (eg, if RSV onset was week 40 in 2019, then the expected RSV onset after the beginning of the COVID-19 was week 40 in 2020). The period at risk for RSV rebound ended either when RSV rebound occurred or when the observation ended (the last week of available RSV data by 8 September 2021), whichever came earlier (Figure 1B).

#### Exposures

We considered several time-dependent exposures that were perceived to be associated with RSV rebound and had available data. In brief, we considered the Google retail and recreation community mobility metric as an objective measure for NPI stringency, and we included climate factors, daily average temperature, relative humidity, and wind speed; a binary indicator of whether countries banned international arrivals from any countries; COVID-19 14-day cumulative notification rate (available on a weekly basis); and school opening status. For school opening status, 3 levels were included: (1) fully open; (2) partially open (defined as open/closed in certain regions only; and/or open/closed for some grade levels/age groups only; and/or open but with reduced in-person class time, combined with distance learning); and (3) closure (as reference). Detailed description of these exposures is shown in Supplementary Table 1.

### Data Analysis

We used a piecewise additive mixed model (PAMM) for the time-to-event analysis [23]. In brief, in PAMM, the observation period is broken down into a finite number of intervals and one assumes that hazard rates are piecewise constant in each of these intervals; then a generalized additive model is applied to estimate the baseline hazard as well as other time-varying covariates semiparametrically. This was done using the R package “pammtools” [24] and “mgcv” [25]. We first considered a complete model with all exposures included, and then the main model was determined through a stepwise backwards variable elimination process from the complete model by comparing model Akaike information criterion (see Supplementary Text). This was to maintain the balance between goodness of fit and parsimony. The complete model is given by:


$$\text{log}(\lambda_{i}(t;x_{ji}))=\beta_{0}+\underset{j=1}{\sum^{J}}\beta_{j}x_{ji}+f\left(t\right),$$

where λ denotes hazard rates; *i* denotes each country; *t* denotes time at risk; *x*_*j*_ denotes the exposure of interest, *j*; and *f* denotes a spline smooth function that will be estimated through restricted maximum likelihood.

As the definition for RSV onset was based on the history of RSV activity for 5 or more weeks, we selected to average the exposures using a 5-week time window before fitting the data into the model. We also applied a time lag of 2 weeks between exposures and outcome considering the possible time lag between RSV infection and reporting. We conducted a series of sensitivity analyses that assessed different RSV definitions, time windows for averaging exposures, and time lags between exposures and outcome; we also conducted an ad hoc sensitivity analysis that used a dichotomous school opening status, school open vs closure (details of all sensitivity analyses are shown in Supplementary Table 2). Furthermore, we conducted an ad hoc exploratory analysis that allowed for time-varying effects and nonlinear effect of temperature (through a spline smooth function); based on this model, we predicted the risk for RSV rebound for the first 10 weeks after schools fully reopen or close at different times (relative to the expected RSV onset) as well as the risk for RSV rebound when schools remain fully open or closed, with varied temperatures.

All statistical analyses and visualizations were conducted using the R software package (version 4.0.5).

## RESULTS

### Countries Included

All 18 countries included in the analysis experienced delayed RSV onset. Eleven countries (61%) observed RSV rebound based on data available by 8 September 2021; compared with the expected RSV onset, RSV rebound was delayed by a range of 5–54 weeks (Table 1). Detailed country-specific data on changes in the exposures of interest over time are shown in Supplementary Figures 1 and 2.

**Table 1.** Overview of Countries Included in the Analysis

Country	Start of Period at Risk for RSV Rebound (T1)	RSV Rebound	End of Period at Risk for RSV Rebound (T2)	Duration of Period at Risk for RSV Rebound, Weeks (T2 − T1)
Australia	Week 15, 2020^a^	Yes	Week 38, 2020	23
Belgium	Week 45, 2020	Yes	Week 8, 2021	16
Canada	Week 42, 2020	Yes	Week 28, 2021	27
Chile	Week 25, 2020	No	Week 27, 2021	62
Denmark	Week 46, 2020	No	Week 20, 2021	34
England	Week 43, 2020	Yes	Week 22, 2021	32
France	Week 45, 2020	Yes	Week 4, 2021	12
Iceland	Week 4, 2021	Yes	Week 9, 2021	5
Ireland	Week 44, 2020	No	Week 30, 2021	39
Japan	Week 26, 2020	Yes	Week 6, 2021	33
Netherlands	Week 50, 2020	Yes	Week 26, 2021	29
New Zealand	Week 24, 2020	Yes	Week 25, 2021	54
Paraguay	Week 21, 2020	No	Week 22, 2021	62
Portugal	Week 49, 2020	No	Week 20, 2021	30
Slovenia	Week 49, 2020	Yes	Week 20, 2021	32
South Korea	Week 41, 2020	No	Week 29, 2021	49
Spain	Week 49, 2020	Yes	Week 20, 2021	30
Sweden	Week 47, 2020	No	Week 20, 2021	26

The period at risk for RSV rebound started at the expected week of RSV onset based on the 2019 data and ended at the week of RSV rebound or the last week of the latest RSV reports (that were available by 8 September 2021), whichever came earlier.

Abbreviation: RSV, respiratory syncytial virus.

RSV season had already started in Australia in the beginning of 2020 until being interrupted by the coronavirus disease 2019 pandemic; we selected the week when RSV season was interrupted as the start of the period at risk for Australia.

### Drivers of RSV Rebound

**Figure 2.**
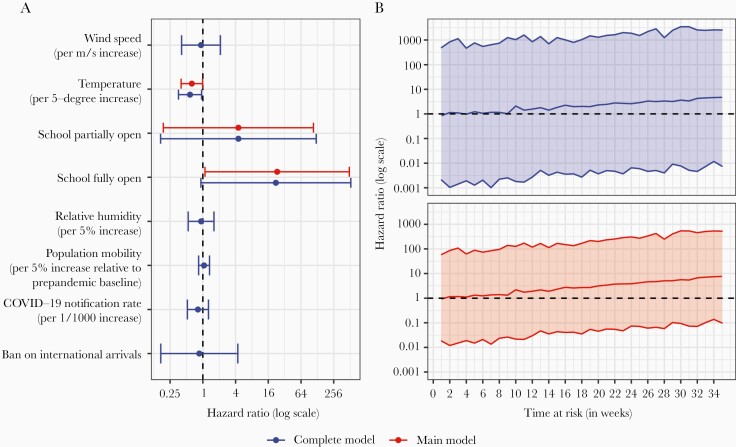
Effect of time-dependent exposures (*A*) and time at risk (*B*) on respiratory syncytial virus rebound. *A*, Dots denote the point estimates and error bars denote the corresponding 95% confidence intervals (CIs). *B*, Reference is the starting week of observation (ie, week 0); lines in the middle denote the point estimates and upper and lower lines denote the corresponding 95% CIs. Abbreviation: COVID-19, coronavirus disease.

From the complete model that included all exposures of interest, we found that both partial and full (re)opening of schools might increase the risk for RSV rebound, although the hazard ratio (HR) was not statistically significant for either. As an independent factor, increased temperatures could reduce the risk (HR, 0.58 [95% confidence interval {CI}, .36–.95] for every 5°C increase). Other factors did not apparently have an effect on the risk for RSV rebound (Figure 2A).

Our main model, selected through the backwards model selection process, showed that full (re)opening of schools was associated with an increased risk for RSV rebound (HR, 23.29 [95% CI, 1.09–495.84]) and that every 5°C increase in temperature was associated with a decreased risk for RSV rebound (HR, 0.63 [95% CI, .40–.99]). Partial (re)opening of schools was not found to be associated with the risk for RSV rebound (Figure 2A). Moreover, there was an increasing trend in the risk for RSV rebound over time since the expected RSV onset in the 2020 or 2020–2021 season, from both the complete and main models (Figure 2B).

### Sensitivity Analysis

Results from predefined sensitivity analyses generally confirmed the findings above. Notably, the findings were sensitive to a less specific definition for RSV onset (ie, 4 net increasing weeks rather than 5 in the main analysis, which resulted in 2 more countries having RSV rebound [Denmark and Portugal]). The results from the ad hoc sensitivity analysis that used a dichotomous school opening status showed statistically nonsignificant HR estimates for school reopening (Supplementary Figure 3).

### Different Scenarios on School Opening and Risk for RSV Rebound

**Figure 3.**
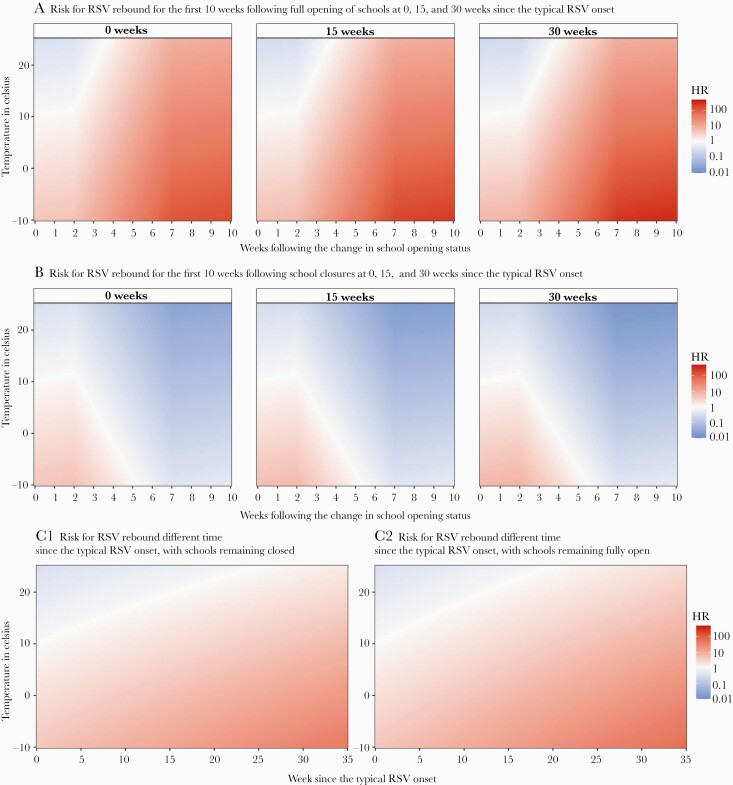
Predicted risk for respiratory syncytial virus (RSV) rebound under different scenarios on school opening status. A 2-week time lag in the effect of school opening/closing was assumed. For all comparisons, reference temperature was set as 10°C (the median temperature when a typical RSV season occurs in the 18 countries) and reference week was week 0 (ie, the week when school opening status changes for panels *A* and *B*, and the week of typical RSV onset for panel *C*.) Abbreviations: HR, hazard ratio; RSV, respiratory syncytial virus.

Furthermore, we assessed the risks for RSV rebound in the first 10 weeks following full (re)opening of schools or school closures. Full (re)opening of schools could substantially increase the risk for RSV rebound, particularly with decreased temperatures and even at high temperatures (Figure 3A). Closing schools (from fully open) could gradually decrease the risk for RSV rebound although to a lesser extent when temperatures decrease (Figure 3B). The risk for RSV rebound also increased over time (since the expected typical RSV onset) even at high temperatures when schools remain closed or fully open (Figure 3C.

## DISCUSSION

Our findings suggest that full (re)opening of schools is the predominant risk factor for RSV rebound, increasing the risk for RSV rebound by as much as 23-fold (95% CI, 1.09- to 459.84-fold). High temperature decreases the risk for RSV rebound, with every 5°C increase reducing the risk by 37% (95% CI, 1%–60%). The risk for RSV rebound also increases over time since the expected typical RSV onset, highlighting the role of the increased susceptible population. Our scenario analysis suggests that full (re)opening of schools can substantially increase the risk for RSV rebound when temperature drops and still increase the risk even at high temperatures. Growing susceptibility and full (re)opening of schools could both override the countereffect of high temperatures, which explains the out-of-season RSV epidemics during the COVID-19 pandemic. Based on empirical data, these findings provide timely evidence-based recommendation for the prevention and control of RSV epidemics in the context of COVID-19 pandemic.

The predominant role of full (re)opening of schools in RSV rebound highlighted in our study is consistent with the findings from a household cohort study in Kenya [26], which suggests that school-aged children play an important role in the spread of RSV, especially to infants within the family who are most vulnerable to developing severe RSV disease. Second to full (re)opening of schools, high temperature could decrease the risk for RSV rebound, which aligns well with its typical season in most temperate countries [4]. Moreover, our findings reveal a continuously increasing trend in the risk for RSV rebound over time. This is likely a result of the increase in the RSV-susceptible population over time, due to the growing number of newborns after the COVID-19 pandemic who remain naive to RSV as well as the buildup of the number of older children who were not infected by RSV in early infancy, including the school-aged children who play an important role in RSV transmission.

Our scenario analysis suggests that countries in the northern hemisphere that have not observed RSV rebound and therefore have a larger than normal susceptible population might expect RSV rebound soon if schools fully reopen in fall 2021. Health systems in these countries should prepare for a surge in RSV cases that might happen even earlier than their typical RSV season. Our scenario analysis also suggests that school reopening could substantially increase the risk for RSV rebound even at high temperatures. This could help explain the delay in out-of-season RSV rebound observed in some countries, such as the United Kingdom.

We acknowledge several caveats to interpreting these results. First, while we identified school opening and temperature as important drivers, we might lack the statistical power to rule out other exposures of interest as important risk/protective factors. One example is ban on international arrivals. International travels declined substantially following the COVID-19 pandemic, which slowed the global seeding of RSV and might have delayed the normal RSV season. This is supported by a recent study in Australia by Eden and colleagues [17], which revealed a significant reduction in RSV genetic diversity following the COVID-19 pandemic. Another example is concurrent SARS-CoV-2 activity. Viral interference could play a role in the delayed RSV onset. A recent systematic analysis [20] showed that the 2009 influenza pandemic, in which widescale NPIs were not employed, delayed RSV onset on average by 0.58 months, suggesting possible viral interference between the pandemic influenza strain and RSV. More generally, viral interference could also explain why some viruses, such as rhinoviruses [10, 27, 28], restored circulation early after NPIs were relaxed, whereas the activity of other viruses such as influenza virus [15, 29] remained low. However, we were unable to include these viruses in our model due to the absence of accessible data.

Second, we focused our analysis on the timing of RSV rebound; due to data scarcity, we were unable to evaluate how different factors could affect the magnitude or severity profile of RSV rebound. A modeling study using pre–COVID-19 pandemic RSV data by Baker and colleagues predicted that future RSV rebound would occur with higher-than-usual magnitude [18]. However, RSV rebound with lower-than-usual magnitude was observed in countries such as the United States and France [30]. A better understanding of how future RSV epidemics would evolve requires the continuation of RSV surveillance, which was interrupted in multiple sites during the COVID-19 pandemic. We were also unable to stratify our analysis by age group due to data scarcity; studies from Australia [31] and France [32] both suggest that compared with the prepandemic period, children hospitalized for RSV were significantly older during the COVID-19 pandemic. For school opening, due to the absence of relevant data, we could not further assess the effect of opening of different grades (eg, primary vs secondary) that are expected to drive RSV transmission differently. We were also unable to consider the opening of preschool facilities (eg, daycare centers) due to the absence of relevant data.

Third, in addition to school closure and international travel bans as individual NPIs, we attempted to use the Google retail and recreation mobility as an objective measure for other NPIs (eg, limits on visits to restaurants, cinemas, shopping malls) considering that the contexts of these NPIs were often fully comparable among different countries. As a result, we were unable to separate the NPIs out in our study. We were also unable to fully account for several NPIs, such as wearing of face-covering and social distancing, that could not be captured well by the mobility data.

Fourth, we only selected 18 countries that had available RSV surveillance data and data on all exposures of interest. Tropical countries were underrepresented in our analysis. A recent study from Thailand observed a delay of about 2 months in the RSV season [10]. Last, we were unable to account for any changes in healthcare-seeking behaviors and healthcare practices since the COVID-19 pandemic, which might contribute to a short-term delay in RSV reporting.

By breaking the longstanding periodicity of RSV activity, the ongoing COVID-19 pandemic, as well as the public health responses to it, offers a unique opportunity to disentangle different factors that could affect RSV transmission dynamics. Our study highlights full (re)opening of schools and growing population susceptibility as the predominant drivers for RSV rebound that could override the countereffect of high temperatures. Our findings could help explain the seasonal RSV epidemics observed in every fall (when schools are opened and temperature drops) in most temperate countries. These findings have important implications for countries’ preparedness for RSV rebound and shed light on the mystery of the mechanism of RSV seasonality. Although it remains unknown whether RSV will return to its pre–COVID-19 pandemic seasonality, experience from the previous 2009 influenza pandemic suggests that the RSV season restored to normality 1 year after the pandemic [20]. It will be important to continue, or in some cases reestablish, surveillance for RSV at this stage of the COVID-19 pandemic to better understand the epidemiology of RSV transmission as well as prepare for the burden of RSV rebound on the public health system.

## Supplementary Data

Supplementary materials are available at *The Journal of Infectious Diseases* online. Supplementary materials consist of data provided by the author that are published to benefit the reader. The posted materials are not copyedited. The contents of all supplementary data are the sole responsibility of the authors. Questions or messages regarding errors should be addressed to the author.

jiab606_suppl_Supplementary_Appendix

## Notes

***Author contributions*.** Y. L. and X. W. conceptualized the study; Y. L. led data acquisition with substantial contribution from B. C. and S. D.; Y. L. led data analysis and visualization with substantial contribution from X. W.; Y. L. wrote the draft report; and all authors revised the report critically for important intellectual content. All authors have read and approved the final version of the report. Y. L., B. C., and S. D. had access to the study data, and the corresponding author (Y. L.) had final responsibility for the decision to submit for publication.

***Acknowledgments.*** Y. L., X. W., and H. N. are members of the Respiratory Syncytial Virus Consortium in Europe, which has received funding from the Innovative Medicines Initiatives (IMI) 2 Joint Undertaking (grant number 116019). This Joint Undertaking receives support from the European Union’s Horizon 2020 Research and Innovation program and the European Federation of Pharmaceutical Industries and Associations.

***Data sharing.*** All data used in this study are publicly available and have been cited in the article or the Supplementary Materials.

***Financial support.*** This work was supported by the Bill & Melinda Gates Foundation and the World Health Organization (WHO).

***Potential conflicts of interest.*** Y. L. and H. N. report grants from the WHO relating to the current study. Y. L. reports grants from the Wellcome Trust outside the submitted work. H. N. reports grants from the IMI; consulting fees from the Bill & Melinda Gates Foundation, Pfizer, and Sanofi; honoraria from AbbVie; support from Sanofi for attending meetings; and participation on advisory boards from Sanofi, Janssen, Novavax, Reviral, Resvinet, and the WHO, outside the submitted work. All other authors report no potential conflicts of interest.
