# Supplementary material for: Understanding the Potential Drivers for Respiratory Syncytial Virus Rebound During the Coronavirus Disease 2019 Pandemic
Source: J Infect Dis. 2022 Jan 14;225(6):957–64. doi: 10.1093/infdis/jiab606 (PMC8807230; doi:10.1093/infdis/jiab606)
Supplement: jiab606_suppl_Supplementary_Appendix [file jiab606_suppl_supplementary_appendix.docx]

Contents

[Table S1. Summary of data sources 2](#_Toc83022631)

[Table S2. Summary of sensitivity analyses 4](#_Toc83022632)

[Supplementary Text - stepwise model selection 5](#_Toc83022633)

[Figure S1. Changes over time in exposures of interest among countries that observed RSV rebound 7](#_Toc83022634)

[Figure S2. Changes over time in exposures of interest among countries that did not observe RSV rebound by end of observation 8](#_Toc83022635)

[Figure S3. Results of sensitivity analyses 9](#_Toc83022636)

[STROBE checklist 10](#_Toc83022637)

# Table S1. Summary of data sources

| Category | Source | Countries | Description |
| --- | --- | --- | --- |
| RSV | New South Wales Government. [COVID-19 weekly surveillance in NSW](https://www.health.nsw.gov.au/Infectious/covid-19/Pages/weekly-reports.aspx). (Last access: 8-September-2021) | Australia | Provides weekly RSV activity since 2020, with comparison to 2016–19. |
| RSV | Sciensano. [Weekly bulletin respiratory infections](https://epidemio.wiv-isp.be/ID/diseases/SiteAssets/Pages/Influenza/WeeklyBulletinRespiratoryInfections.pdf). (Last access: 8-September-2021) | Belgium | Provides weekly RSV activity for the recent five years. |
| RSV | Public Health Agency of Canada. [Respiratory Virus Detections in Canada](https://www.canada.ca/en/public-health/services/surveillance/respiratory-virus-detections-canada.html). (Last access: 8-September-2021) | Canada | Provides weekly RSV activity between 2013 and 2021. Only national data were included for the analysis. |
| RSV | PAHO. [SARI surveillance.](http://ais.paho.org/phip/viz/flumart2015.asp) (Last access: 8-September-2021) | Chile, Paraguay | Provides weekly RSV activity from SARI surveillance for most countries in Latin America. Data availability depends on countries and years. Only countries with complete data between 2019 and 2021 were considered for the analysis. |
| RSV | ECDC. [Surveillance Atlas of Infectious Diseases](https://atlas.ecdc.europa.eu/public/index.aspx). (Last access: 8-September-2021) | Denmark, France, Iceland, Ireland, Portugal, Slovenia, Spain, Sweden | Provides weekly RSV activity for countries in EU/EEA. Data availability depends on countries and years. Only countries with complete data between 2019 and 2021 were considered for the analysis. |
| RSV | Public Health England, UK. [National flu and COVID-19 surveillance reports](https://www.gov.uk/government/statistics/national-flu-and-covid-19-surveillance-reports). (Last access: 8-September-2021) | England | Provides weekly RSV activity between 2018 and 2021. |
| RSV | Japan National Institute of Infectious Diseases. [Infectious Diseases Weekly Report](https://www.niid.go.jp/niid/en/10/2096-weeklygraph/1661-21rsv.html). (Last access: 8-September-2021) | Japan | Provides weekly RSV activity between 2018 and 2021. |
| RSV | RIVM. [State of affairs RS virus.](https://www.rivm.nl/rs-virus/stand-van-zaken-rs-virus) (Last access: 8-September-2021) | Netherlands | Provides weekly RSV activity between 2015 and 2021. |
| RSV | The Institute of Environmental Science and Research. [Flu surveillance and research.](https://www.esr.cri.nz/our-services/consultancy/flu-surveillance-and-research/) (Last access: 8-September-2021) | New Zealand | Provides weekly RSV activity between 2019 (from archived dashboard) and 2021. |
| RSV | Korea Disease Control and Prevention Agency. [Clinical sentinel surveillance system - acute respiratory infection.](http://www.kdca.go.kr/npt/biz/npp/iss/ariStatisticsMain.do) (Last access: 8-September-2021) | South Korea | Provides weekly RSV activity between 2015 and 2021. |
| Population mobility | Google LLC. [COVID-19 Community Mobility Reports.](https://www.google.com/covid19/mobility/) (Last access: 8-September-2021) | Over 130 countries | Provides daily mobility changes compared to the pre-pandemic baseline (3-January-2020 to 6-February-2020). Six mobility metrics are available: retail and recreation, grocery and pharmacy, workplaces, parks, public transit, and residential. The mobility metrics of retail and recreation were included in the analysis as an objective measure of the stringency of NPIs. |
| NPI | [Oxford COVID-19 Government Response Tracker.](https://www.bsg.ox.ac.uk/research/research-projects/covid-19-government-response-tracker) (Last access: 8-September-2021) | Over 180 countries | Tracks daily policy responses to COVID-19 since 1-January-2020, which are coded into 23 indicators. The indicator of international travel ban was used and re-coded in the analysis. |
| School opening status | UNESCO. [COVID-19 impact on education.](https://en.unesco.org/covid19/educationresponse#schoolclosures) (Last access: 8-September-2021) | Over 200 countries | Provides daily school opening status since 17-February-2020, which has four levels:  Closed due to COVID-19, academic break, partially open (defined as (**a**) open/closed in certain regions only; and/or (**b**) open/closed for some grade levels/age groups only; and/or (**c**) open but with reduced in-person class time, combined with distance learning), and fully open. |
| Climate | NOAA. [Global Surface Summary of the Day.](https://www.ncei.noaa.gov/access/metadata/landing-page/bin/iso?id=gov.noaa.ncdc:C00516) (Last access: 8-September-2021) | Over 200 countries | Provides daily weather data from over 9000 weather stations since 1929. |
| COVID-19 | ECDC. [Data on 14-day notification rate of new COVID-19 cases and deaths](https://www.ecdc.europa.eu/en/publications-data/data-national-14-day-notification-rate-covid-19). (Last access: 8-September-2021) | Over 200 countries | Provides weekly data on 14-day notification rate of COVID-19 cases. |

# Table S2. Summary of sensitivity analyses

| Index | Time-varying exposures | Definition for RSV onset/rebound | Time window for averaging exposures | Time lag between exposure and outcome |
| --- | --- | --- | --- | --- |
| Main model | School open (fully open, partially open, closure) + Temperature | 5 net increasing weeks | 5 weeks | 2 weeks |
| Sensitivity 1 | School open (fully open, partially open, closure) + Temperature | 4 net increasing weeks | 5 weeks | 2 weeks |
| Sensitivity 2 | School open (fully open, partially open, closure) + Temperature | 6 net increasing weeks | 5 weeks | 2 weeks |
| Sensitivity 3 | School open (fully open, partially open, closure) + Temperature | 5 net increasing weeks | 3 weeks | 2 weeks |
| Sensitivity 4 | School open (fully open, partially open, closure) + Temperature | 5 net increasing weeks | 7 weeks | 2 weeks |
| Sensitivity 5 | School open (fully open, partially open, closure) + Temperature | 5 net increasing weeks | 5 weeks | 1 week |
| Sensitivity 6 | School open (fully open, partially open, closure) + Temperature | 5 net increasing weeks | 5 weeks | 3 weeks |
| Sensitivity 7 (ad-hoc) | School open (fully/partially open, closure) + Temperature | 5 net increasing weeks | 5 weeks | 2 weeks |

Texts in red denote the differences from the main model. Results of sensitivity analyses are in Figure S3.

# Supplementary Text - stepwise model selection

Each step of the backwards model selection is detailed below. The final model (after step 5) contains two exposures of interest: school opening status and temperature. For each step, the AIC in red indicates the lowest value (i.e. the best model).

**Complete model**: RSV_rebound ~ s(time) + school + mobility + COVID + temperature + relative_humidity + wind_speed + international_arrival_ban

Complete model: AIC = 111.48

Remove school: AIC = 113.70

Remove mobility: AIC = 109.69

Remove COVID: AIC = 110.54

Remove temperature: AIC = 114.23

Remove relative_humidity: AIC = 109.55

Remove wind_speed: AIC = 109.518

Remove international_arrival_ban: AIC = 109.517

**Best model after step 1**: RSV_rebound ~ s(time) + school + mobility + COVID + temperature + relative_humidity + wind_speed

Best model after step 1: AIC = 109.52

Remove school: AIC = 112.28

Remove mobility: AIC = 107.70

Remove COVID: AIC = 108.56

Remove temperature: AIC = 112.27

Remove relative_humidity: AIC = 107.61

Remove wind_speed: AIC = 107.57

**Best model after step 2**: RSV_rebound ~ s(time) + school + mobility + COVID + temperature + relative_humidity

Best model after step 2: AIC = 107.57

Remove school: AIC = 110.42

Remove mobility: AIC = 105.76

Remove COVID: AIC = 106.56

Remove temperature: AIC = 110.45

Remove relative humidity: AIC = 105.82

**Best model after step 3**: RSV_rebound ~ s(time) + school + COVID + temperature + relative humidity

(to be continued)

Best model after step 3: AIC = 105.76

Remove school_fullyopen: AIC = 108.61

Remove COVID: AIC = 105.38

Remove temperature: AIC = 108.45

Remove wind_speed: AIC = 103.97

**Best model after step 4**: RSV_rebound ~ s(time) + school + COVID + temperature

Best model after step 4: AIC = 103.97

Remove school: AIC = 106.67

Remove COVID: AIC = 103.92

Remove temperature: AIC = 106.46

**Best model after step 5**: RSV_rebound ~ s(time) + school + temperature

Best model after step 5: AIC = 103.92

Remove school: AIC = 108.29

Remove temperature: AIC = 106.12

# Figure S1. Changes over time in exposures of interest among countries that observed RSV rebound


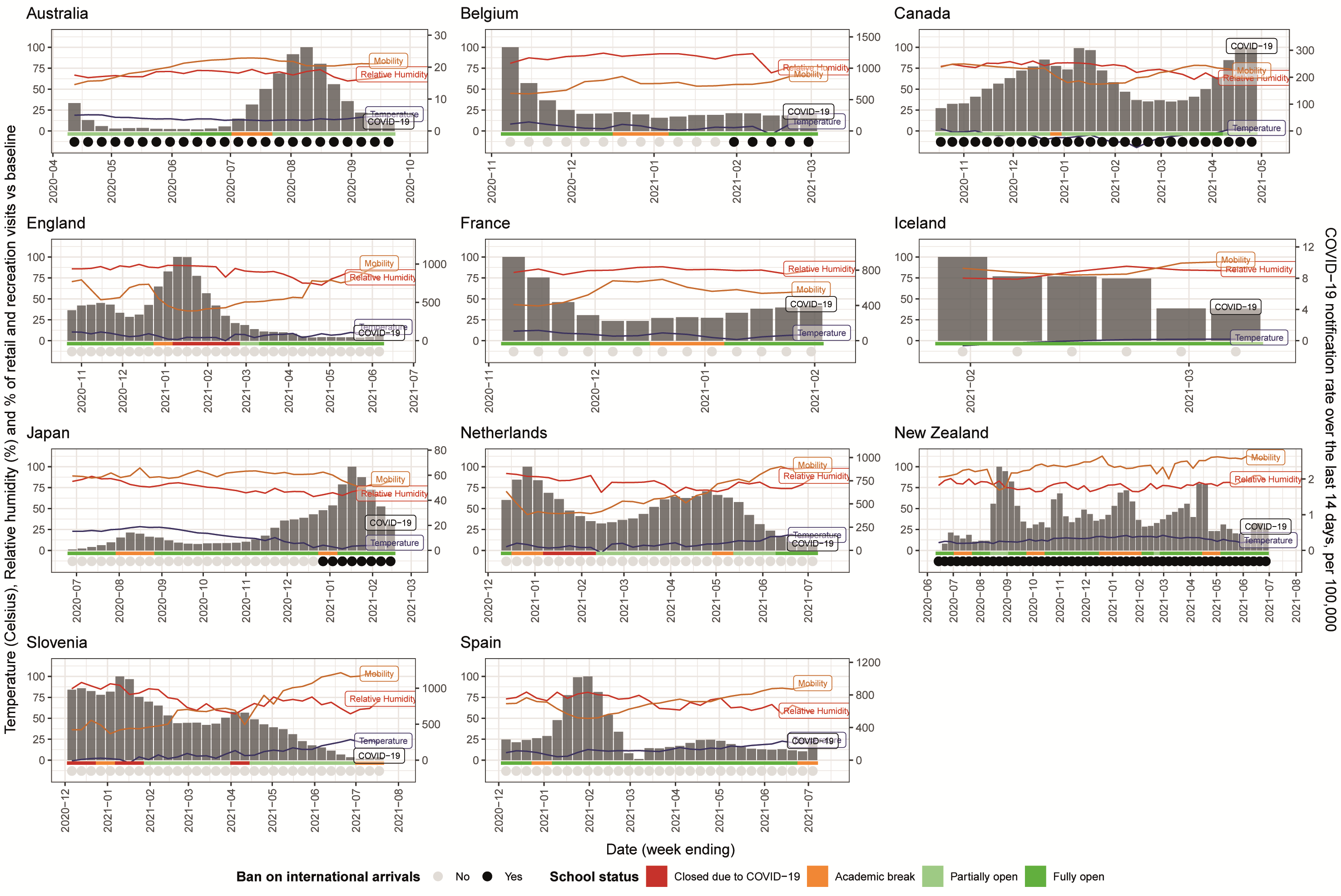


# Figure S2. Changes over time in exposures of interest among countries that did not observe RSV rebound by end of observation


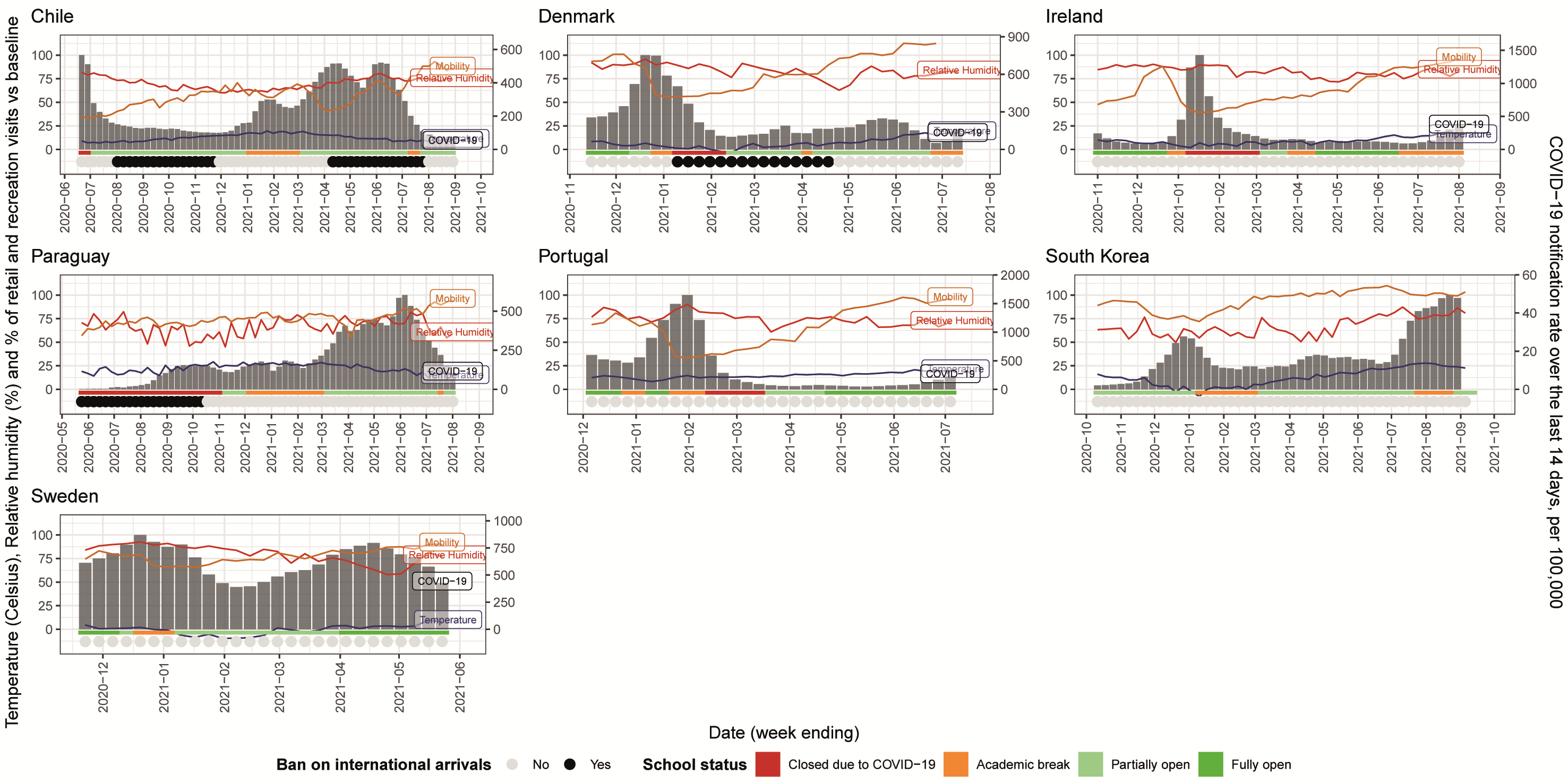


# Figure S3. Results of sensitivity analyses


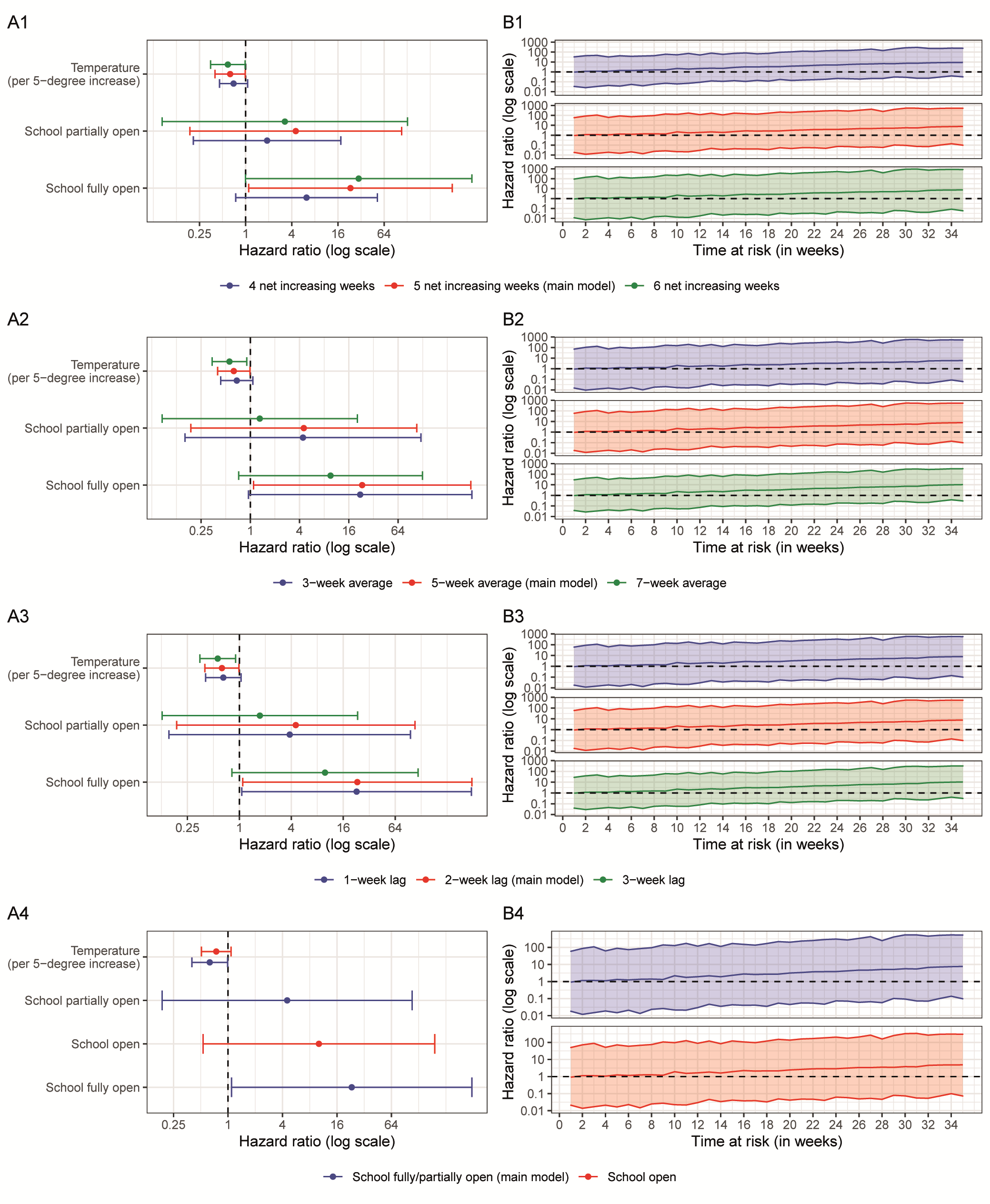


# STROBE checklist

STROBE Statement—checklist of items that should be included in reports of observational studies

|  | Item No | Recommendation | Page No |
| --- | --- | --- | --- |
| **Title and abstract** | 1 | (*a*) Indicate the study’s design with a commonly used term in the title or the abstract | 1 |
|  |  | (*b*) Provide in the abstract an informative and balanced summary of what was done and what was found |  |
| Introduction | | |  |
| Background/rationale | 2 | Explain the scientific background and rationale for the investigation being reported | 3 |
| Objectives | 3 | State specific objectives, including any prespecified hypotheses | 3 |
| Methods | | |  |
| Study design | 4 | Present key elements of study design early in the paper | 3 |
| Setting | 5 | Describe the setting, locations, and relevant dates, including periods of recruitment, exposure, follow-up, and data collection | 3 |
| Participants | 6 | (*a*) *Cohort study*—Give the eligibility criteria, and the sources and methods of selection of participants. Describe methods of follow-up  *Case-control study*—Give the eligibility criteria, and the sources and methods of case ascertainment and control selection. Give the rationale for the choice of cases and controls  *Cross-sectional study*—Give the eligibility criteria, and the sources and methods of selection of participants | 3 |
|  |  | (*b*) *Cohort study*—For matched studies, give matching criteria and number of exposed and unexposed  *Case-control study*—For matched studies, give matching criteria and the number of controls per case |  |
| Variables | 7 | Clearly define all outcomes, exposures, predictors, potential confounders, and effect modifiers. Give diagnostic criteria, if applicable | 3-4 |
| Data sources/ measurement | 8* | For each variable of interest, give sources of data and details of methods of assessment (measurement). Describe comparability of assessment methods if there is more than one group | 3-4 |
| Bias | 9 | Describe any efforts to address potential sources of bias | 5 |
| Study size | 10 | Explain how the study size was arrived at | 3-4 |
| Quantitative variables | 11 | Explain how quantitative variables were handled in the analyses. If applicable, describe which groupings were chosen and why | 3-4 |
| Statistical methods | 12 | (*a*) Describe all statistical methods, including those used to control for confounding | 5-6 |
|  |  | (*b*) Describe any methods used to examine subgroups and interactions |  |
|  |  | (*c*) Explain how missing data were addressed |  |
|  |  | (*d*) *Cohort study*—If applicable, explain how loss to follow-up was addressed  *Case-control study*—If applicable, explain how matching of cases and controls was addressed  *Cross-sectional study*—If applicable, describe analytical methods taking account of sampling strategy |  |
|  |  | (*e*) Describe any sensitivity analyses |  |

Continued on next page

| Results | | |  |
| --- | --- | --- | --- |
| Participants | 13* | (a) Report numbers of individuals at each stage of study—eg numbers potentially eligible, examined for eligibility, confirmed eligible, included in the study, completing follow-up, and analysed | 6 |
|  |  | (b) Give reasons for non-participation at each stage |  |
|  |  | (c) Consider use of a flow diagram |  |
| Descriptive data | 14* | (a) Give characteristics of study participants (eg demographic, clinical, social) and information on exposures and potential confounders | Figures S1 & S2 |
|  |  | (b) Indicate number of participants with missing data for each variable of interest |  |
|  |  | (c) *Cohort study*—Summarise follow-up time (eg, average and total amount) |  |
| Outcome data | 15* | *Cohort study*—Report numbers of outcome events or summary measures over time | Table 1 |
|  |  | *Case-control study—*Report numbers in each exposure category, or summary measures of exposure |  |
|  |  | *Cross-sectional study—*Report numbers of outcome events or summary measures |  |
| Main results | 16 | (*a*) Give unadjusted estimates and, if applicable, confounder-adjusted estimates and their precision (eg, 95% confidence interval). Make clear which confounders were adjusted for and why they were included | 6-7 |
|  |  | (*b*) Report category boundaries when continuous variables were categorized |  |
|  |  | (*c*) If relevant, consider translating estimates of relative risk into absolute risk for a meaningful time period |  |
| Other analyses | 17 | Report other analyses done—eg analyses of subgroups and interactions, and sensitivity analyses | 7 |
| Discussion | | |  |
| Key results | 18 | Summarise key results with reference to study objectives | 7 |
| Limitations | 19 | Discuss limitations of the study, taking into account sources of potential bias or imprecision. Discuss both direction and magnitude of any potential bias | 8-9 |
| Interpretation | 20 | Give a cautious overall interpretation of results considering objectives, limitations, multiplicity of analyses, results from similar studies, and other relevant evidence | 8 |
| Generalisability | 21 | Discuss the generalisability (external validity) of the study results | 9 |
| Other information | | |  |
| Funding | 22 | Give the source of funding and the role of the funders for the present study and, if applicable, for the original study on which the present article is based | 11 |

*Give information separately for cases and controls in case-control studies and, if applicable, for exposed and unexposed groups in cohort and cross-sectional studies.

**Note:** An Explanation and Elaboration article discusses each checklist item and gives methodological background and published examples of transparent reporting. The STROBE checklist is best used in conjunction with this article (freely available on the Web sites of PLoS Medicine at http://www.plosmedicine.org/, Annals of Internal Medicine at http://www.annals.org/, and Epidemiology at http://www.epidem.com/). Information on the STROBE Initiative is available at www.strobe-statement.org.
